# Age at Onset of Declarative Gestures and 24-Month Expressive Vocabulary Predict Later Language and Intellectual Abilities in Young Children With Williams Syndrome

**DOI:** 10.3389/fpsyg.2019.02648

**Published:** 2019-12-03

**Authors:** Angela M. Becerra, Carolyn B. Mervis

**Affiliations:** Department of Psychological and Brain Sciences, University of Louisville, Louisville, KY, United States

**Keywords:** child gesture, lexical development, language development, early predictors, Williams syndrome, intellectual disability, longitudinal

## Abstract

**Background:** One of the most consistent findings in the early language acquisition literature regarding children in the general population is that the onset of declarative pointing gestures precedes the onset of expressive referential language. Furthermore, frequency of early use of declarative gestures is a stronger predictor of later lexical development than early vocabulary size. These findings suggest that early declarative gestures may play a critical facilitative role in later language development. To evaluate the universality of these findings, we tested children with Williams syndrome (WS), a genetic disorder associated with both language and communicative gesture delay.

**Method:** Participants were 47 children with classic-length WS deletions. Age of onset of declarative *show* and *point* were determined by parental report on the MacArthur-Bates Communicative Development Inventory (CDI): Words and Gestures. Expressive vocabulary size at onset of these gestures, first referential expressive word, 24 months, and 48 months and grammatical complexity at 48 months were determined by parental report on the CDI: Words and Sentences. Receptive and expressive vocabulary and overall intellectual ability at 48 months were measured using standardized assessments.

**Results:** In contrast to previous findings for children in the general population, most children with WS began to produce referential language several months before they produced declarative *point*. A series of multiple regressions indicated that both age at onset of declarative *point* and expressive vocabulary size at 24 months made significant independent contributions to individual differences in lexical, grammatical, and overall intellectual ability at 48 months. Similarly to the findings for typically developing children and children with other developmental disabilities, individual differences in the declarative gesture measure accounted for considerably more variance in 48-month lexical ability than early expressive vocabulary size.

**Discussion:** The transition from prelinguistic communication to initial referential language does not depend on the onset of the ability to use declarative *point*. Nevertheless, the length of time that a child has been producing declarative *point* was a better predictor than early expressive vocabulary of later lexical abilities. Thus, despite the earlier divergence in their path to language development from the typical one, the path for children with WS re-converges with that for typically developing children and children with other developmental disabilities.

## Introduction

Since the pioneering work of Elizabeth Bates and her colleagues (e.g., Bates et al., 1979) on the relations between early communicative gesture development and early lexical development, a growing body of research has demonstrated consistent relations between the onset of declarative (referential) gesture acquisition and the onset of expressive referential language. Furthermore, early declarative gesturing ability has been shown to be strongly related not only to concurrent expressive vocabulary size but also to vocabulary ability months or even a few years later. This body of research has focused primarily on typically developing (TD) children. To determine if the relations found for TD children are universal, studies of children with developmental disabilities (DD) are crucial. To date, such studies have focused primarily on children with autism spectrum disorder (ASD) or Down syndrome (DS). In the present study, we address these relations for children with Williams syndrome (WS).

WS is a neurodevelopmental disorder caused by a hemizygous microdeletion of 26–28 genes on chromosome 7q11.23 (Hillier et al., 2003), with an estimated prevalence of 1 in 7,500 live births (Strømme et al., 2002). Individuals with WS typically have overall intellectual abilities in the borderline to moderate intellectual disability range with a characteristic pattern of relative strength in verbal and nonverbal reasoning and severe weakness in visuospatial construction (Mervis et al., 2000; Mervis and John, 2010). Within the verbal domain, the areas of greatest strength are concrete vocabulary and phonological processing and the area of greatest weakness is relational vocabulary (Mervis, 2009; Mervis and John, 2010). Despite the fact that language abilities are a relative strength for individuals with WS, the onset of language is delayed and the rate of vocabulary acquisition once language development begins is slower than expected for TD children (Mervis and Robinson, 2000; Mervis and Becerra, 2007; Mervis and John, 2012). The purpose of the present study was two-fold: (1) to determine the temporal relation between the onset of declarative gestures and the onset of referential expressive language for very young children with WS and (2) to determine the predictive validity of individual differences in chronological age (CA) at the onset of declarative pointing and expressive vocabulary size at 24 months for individual differences in later vocabulary and intellectual abilities for children with this syndrome.

### Relation Between the Onset of Referential *Show* and *Point* and the Onset of Referential Expressive Language

Researchers studying children’s pointing and showing gestures typically differentiate between two types: declarative and imperative. Gestures are considered “declarative” when the child’s goal is simply to direct another person’s attention to something in the environment that the child considers to be interesting. In contrast, gestures are considered “imperative” when the child’s intent is to direct another person’s behavior in order for the child to obtain a goal (e.g., Carpenter et al., 1998). Based on a meta-analysis, Colonnesi et al. (2010) have shown that the development of referential expressive language is related to declarative pointing but not to imperative pointing.

The sequential relation between the onsets of referential pointing and referential naming, with referential pointing being acquired prior to referential naming, is one of the most robust findings in the early communicative development literature. This sequential order was routinely mentioned in textbooks on early communicative development (e.g., Messer, 1994; Adamson, 1995) and has been found for TD children acquiring not only spoken American English but also a variety of European spoken and signed languages (see review in Mervis and Bertrand, 1997). In the most recent study to address the relation between the onset of referential gestures and the onset of referential expressive language by TD infants, Carpenter et al. (1998) followed 24 TD infants at monthly intervals from age 9–15 months. All 24 began producing declarative gestures prior to the onset of referential expressive language.

This sequential relation also has been found for children with DS, children with prelingual hearing impairment acquiring signed languages, and children with severe intellectual disability acquiring American English (Mervis and Bertrand, 1997). More recently, this relation has been addressed for toddlers with ASD. In a longitudinal study with monthly data collection, Talbott et al. (in press) found a different pattern than had been reported for TD infants or children with other DDs. In particular, of the 32 participants, only 12 showed the typical pattern of acquisition of distal pointing gestures prior to the onset of expressive language. Of the remaining children, seven first evidenced distal pointing and expressive language during the same month and 13 produced expressive language prior to producing distal pointing gestures.

Most of the studies evaluating the relation between the onset of referential gestures and the onset of referential expressive language have focused on declarative pointing. The studies that have addressed declarative showing have typically combined this gesture with declarative pointing to form a single declarative gesture category. Thus, little information is available about the onset of declarative *show* relative to the onset of declarative *point*. Data from the available studies indicate that on average declarative *show* is acquired about 2 months before declarative *point*. Based on the norming sample for the MacArthur Communicative Development Inventory: Words and Gestures, Fenson et al. (1994) found that the earliest age at which at least 50% of the sample was reported by a parent to produce a particular gesture was 8 months (the youngest age included in the norming sample) for declarative *show* and 10 months for declarative *point*. Carpenter et al. (1998) reported that the mean age at which their participants first produced declarative gestures during their monthly lab visit was 10.7 months for declarative *show* and 12.6 months for declarative *point*.

### Longitudinal Relations Between Quantitative Measures of Declarative Gestures and Vocabulary Size

As part of a series of meta-analyses addressing the relation between pointing and language development for TD infants and toddlers, Colonnesi et al. (2010) considered the longitudinal relation between early declarative pointing gestures and later language. Eleven studies were included, with a range of 2.5–15 months between measurements of the two abilities. The authors reported that the combined effect size was strong for the four studies that considered the relation between production of declarative pointing gestures and later language, *r* = 0.42 [95% CI: 0.26–0.59], and moderate to strong for the seven studies that considered the relation between comprehension of declarative pointing gestures and later language, *r* = 0.38 [95% CI: 0.35–0.51]. More recently, Harbison et al. (2017) conducted a meta-analysis of 23 studies addressing the relation between declarative intentional communicative acts (including pointing gestures) and language ability in children with confirmed ASD diagnoses (The ASD diagnosis could have been determined before, during, or after the study). Both longitudinal studies (with a range of 4–77 months between measurement of intentional communicative acts and measurement of language) and cross-sectional studies were included, yielding a weighted *r* = 0.42 [95% CI: 0.34–0.50]. Neither research design (cross-sectional vs. longitudinal) nor language mode (receptive or expressive) had a significant effect, perhaps due to low power. Together, these findings indicate that for both TD children and children with ASD, early declarative gestural ability is related to later language ability. However, interpretation of this relation is difficult because potential confounding variables (e.g., language ability at the time that gestural ability was measured) were not included in the analyses.

A few longitudinal studies that took into account possible confounding variables have been reported. Rowe et al. (2008), in a study of 52 TD toddlers, found that the number of different meanings conveyed in gesture (number of different objects referenced by a deictic gesture such as pointing or showing plus number of different conventional or representational gestures) at 14 months was strongly correlated with Peabody Picture Vocabulary Test-III (PPVT-III; Dunn and Dunn, 1997) standard score (SS) at 42 months, even after controlling for expressive vocabulary size at 14 months, partial *r* = 0.52. In a multiple regression analysis predicting PPVT-III SS, once number of gesture types at 14 months was added to the regression equation, number of different words at age 14 months no longer made a significant contribution. Findings for the same sample of children when gestures and early expressive vocabulary were measured at 18 months rather than 14 months also indicated a strong effect of number of different gesture meanings on PPVT-III SSs at 42 months, even after controlling for expressive vocabulary size at 18 months (Rowe and Goldin-Meadow, 2009). In the latter analysis, significant effects were found for both number of gesture types at 18 months (82% of which were deictic; see Sauer et al., 2010) and number of word types at 18 months, with the effect for number of gesture types nominally larger than the effect for number of word types.

Three longitudinal studies that included children with developmental disabilities also took into account the possible confounding variable of expressive vocabulary size at the time that gesture ability was measured. Zampini and D’Odorico (2009) measured the number of deictic gestures (pointing or showing) produced by 20 children with DS during a play session at age 36 months and expressive vocabulary size as measured by parental report on Il Primo Vocabolario del Bambino (PVB: Caselli and Casadio, 1995) at both 36 and 42 months. After controlling for expressive vocabulary size at 36 months, the partial correlation between number of deictic gestures at 36 months and expressive vocabulary size at 42 months was *r* = 0.55, indicating a strong longitudinal relation. Sauer et al. (2010) studied 11 children with pre- or perinatal unilateral brain lesions (PL) using the same design as Rowe et al. (2008) and Rowe and Goldin-Meadow (2009). In the multiple regression analysis predicting PPVT-III SS at 30 months, the number of different gesture types (86% of which were deictic) at 18 months contributed a significant amount of unique variance even after the number of word types at 18 months was taken into account. The latter variable did not contribute a significant amount of unique variance to 30-month PPVT-III SS. Manwaring et al. (2019) studied a sample of 92 children [60 TD, 30 with language delay (LD)]. Twelve of the children with LD were diagnosed with ASD at age 30 months. Deictic gesture ability at 18 months was measured by the number of deictic gestures produced during the Communication and Symbolic Behavior Scales Developmental Profile-Behavior Sample (CSBS; Wetherby and Prizant, 2002). Expressive vocabulary was measured by the MacArthur-Bates Communicative Development Inventory (CDI; Fenson et al., 2007) at ages 18 and 36 months. The number of deictic gestures produced during the 18-month CSBS was a significant predictor of 36-month expressive vocabulary, even after taking into account 18-month expressive vocabulary and a series of additional potentially confounding variables. There was no significant effect of group (TD, LD-non-ASD, and LD-ASD) and no significant interactions involving group, perhaps due to low power. In these studies, deictic gestures were not separated into declarative and imperative motives so the possibility that one type of deictic gesture might be a stronger predictor of later expressive language was not addressed. Nevertheless, the findings suggest that the relation of early gestural ability to later expressive language ability likely is not simply a reflection of a strong relation between early and later expressive vocabulary.

### Early Gestural and Lexical Development in Children With Williams Syndrome

The onsets of both expressive language and communicative gestures have consistently been reported to be delayed for children with WS (see Mervis and Becerra, 2007; Mervis and John, 2012 for reviews). Most recently, Becerra (2016) found that of a sample of 49 18-month-olds with WS, 37 (76%) scored at or below the 5th percentile on the expressive vocabulary norms for the CDI: Words and Sentences (CDI-W&S; Fenson et al., 2007). Similarly, 41(83%) of the children scored at or below the 5th percentile on the norms for the Early Gestures Scale of the CDI: Words and Gestures (CDI-W&G; Fenson et al., 2007). Declarative *point* and *show* are both included on the Early Gestures Scale.

The relation between the onset of declarative *point* and the onset of expressive referential language has been examined in one small-sample study of toddlers with WS who were followed monthly. Mervis and Bertrand (1997) reported that nine of the 10 children in their study began to produce referential expressive language prior to beginning to produce referential pointing gestures, with an average of 6 months between the two onsets. The remaining child began to produce referential pointing gestures a few weeks prior to producing his first referential word. Further analyses indicated that for the nine children for whom referential expressive language preceded referential *point*, at the time of the first play session in which the child produced a referential word, none of the children was able to follow the mother’s pointing gestures, indicating that the delay in producing pointing gestures was not due simply to a general delay in motor skills.

Two additional studies provide further evidence of the difficulties young children with WS have with declarative pointing and showing gestures. To provide an overview of the socio-communicative abilities of older toddlers and preschoolers with WS who have limited or no expressive language, Klein-Tasman et al. (2007) administered Module 1 of the Autism Diagnostic Observation Schedule (ADOS; Lord et al., 1999) to 29 participants (mean age 42 months). This module assesses both declarative *point* and declarative *show* abilities. Results indicated that 76% of the children showed abnormalities on the “point” item and 79% evidenced abnormalities on the “show” item. Laing et al. (2002) compared the declarative pointing abilities of older toddlers (mean age 31 months) with WS to those of a group of TD infants (mean age 14 months) matched for mental age. Although all of the children with WS in that study were already producing referential language and the WS group had significantly larger expressive vocabularies than the TD group (mean of 56.0 vs. 31.5 words), the TD group produced significantly more declarative pointing gestures than the WS group (mean of 2.57 vs. 0.08 declarative points) during the Study 1 experimental procedures. These findings were replicated in Study 2, and Study 3 provided evidence that the lack of production of pointing gestures by the children with WS was not due to fine motor problems.

### The Present Study

Two findings consistently emerge from studies of TD children and children with DS addressing the acquisition of referential gestures in relation to the acquisition of referential expressive vocabulary: (1) The onset of referential pointing gestures almost always precedes the onset of referential expressive language. (2) Referential gestural ability early in the language acquisition process predicts expressive vocabulary ability at a later time point. Studies of young children with ASD indicate that although the first finding does not hold for a large proportion of these children, there is some evidence that the second finding does hold.

There has been considerably less research on these topics conducted with children with WS. Research on a small sample indicated that the finding for TD children and children with DS that onset of referential pointing gestures precedes onset of referential expressive language does not hold for most children with WS. However, the relation between the onset of referential *show* – which would be expected to be acquired prior to the onset of referential *point* – and the onset of referential expressive language was not considered. Furthermore, although prior studies (e.g., Mervis and Robinson, 2000; Mervis and John, 2012; Becerra, 2016) have documented wide variation in expressive vocabulary size for young children with WS of a given age, neither the relation between age at onset of referential pointing gestures and expressive vocabulary size at a particular age nor the relation between expressive vocabulary size at one specific age and expressive vocabulary size at a later specific age has been addressed. The purpose of the present study was to address these topics based on a considerably larger sample of children with WS. None of the participants in the present study were included in Mervis and Bertrand (1997). Although 32 of the 18-month-olds in Becerra (2016) were included in the present sample, the analyses described herein are independent of those reported in Becerra (2016). Our first aim was to document the relations between age of acquisition of declarative *show* and declarative *point* and age of onset of referential expressive language. Our second aim was to examine the contributions of individual differences in the age of onset of declarative *point* and individual differences in expressive vocabulary size at 24 months to individual differences in vocabulary ability, grammatical ability, and overall intellectual ability at 48 months.

## Method

### Participants

The present study included 47 children (21 girls and 26 boys) with genetically confirmed classic deletions of the WS region. Mean chronological age (CA) was 24.44 months (SD: 0.29, range: 23.95–24.94) at the 24-month data point and 48.50 months (SD: 0.28, range: 47.97–49.00) at the 48-month data point. All children were native speakers of English; one child also had some exposure to French. The participants’ racial/ethnic background was: 40 White non-Hispanic (85.1%), 2 White Hispanic (4.3%), 1 African-American non-Hispanic (2.1%), and 4 biracial non-Hispanic (8.5%). The sample was geographically diverse: 20 states and the District of Columbia were represented, spanning all four United States Census regions. Data collection began in December 1997 and ended in December 2018.

### Measures

#### Gestural Abilities

Gestural abilities were determined from parental report on the First Communicative Gestures section of the CDI-W&G (Fenson et al., 2007). Age of onset of each of the declarative gestures *show* and *point* was determined based on the child’s CA when the parent first responded either “sometimes” or “often” to the following items: (1) child extends arm to show you something he/she is holding and (2) child points (with arm and index finger extended) at some interesting object or event.

#### Lexical Ability

Throughout the study, lexical ability was determined from parental report on the 680-word Vocabulary Checklist of the CDI-W&S (Fenson et al., 2007). Parents were instructed to mark with an S each word that the child both understood and said spontaneously (not in imitation or as part of a song or other routine), to mark with an M (for “manual sign”) each word that the child both understood and signed spontaneously (not after someone else had said or signed the word and not as part of a song or other routine), and to mark with a B (for “both”) each word that the child understood, said, and signed spontaneously. The child’s expressive vocabulary as measured by the CDI-W&S (hereafter, CDI-EV) was the total number of words the parent marked “S,” “M,” or “B.” CDI-EV was determined monthly. For the present study, CDI-EV at ages 24 and 48 months, at the time of onset of each of the declarative gestures (*show* and *point*), and at the time the child first was reported to spontaneously produce a referential word was determined.

To determine when a child first spontaneously produced a referential word, the word(s) that parents indicated their child said or signed during the first session in which the child was reported to have produced any spontaneous expressive language were classified as either referential or non-referential. Words were considered referential if they referred to people (e.g., mommy), to animals (e.g., kitty) or animal sounds used as animal names (e.g., meow – if used to label a cat), to objects (e.g., ball, banana, and bottle), or to actions (e.g., eat). Words were considered non-referential if they were used as greetings (e.g., hi and bye-bye), as exclamations (e.g., uhoh), or as part of a routine (e.g., more and peekaboo). If a child was not reported to produce at least one referential word during the first session in which he or she produced spontaneous expressive language, then the words that the child produced at subsequent sessions were examined to identify the first session in which he or she was reported to produce at least one word referentially. The age at which the child first was reported to produce a referential word spontaneously was considered the age of onset of referential expressive language.

#### Grammatical Ability

Grammatical ability was determined from parental report on the CDI-W&S Early Sentence Checklist (Fenson et al., 2007). The Early Sentence Checklist is composed of 37 pairs of phrases/sentences covering a variety of grammatical constructions. In each pair, the same concept is presented in a simpler and a more complex form. Parents were asked to indicate which of the two sentences in a pair sounded more like the way their child talked. They were further instructed that if the child did not produce constructions as complex as the first item in the pair, neither item in the pair should be marked; if the child produced constructions whose complexity was between the first and the second items in the pair, the first should be marked; and if the child produced constructions more complex than the second item in the pair, the second item should be marked. The child’s Sentence Complexity score (CDI-SC) was the number of pairs for which the second item was marked. If the parent reported that the child was not combining words spontaneously, the Early Sentence Checklist was not administered and the child’s CDI-SC was recorded as 0. For the present study, CDI-SC at age 48 months was determined.

#### Receptive Vocabulary Ability

Receptive vocabulary ability was measured by the Peabody Picture Vocabulary Test-4 (PPVT-4; Dunn and Dunn, 2007). The PPVT-4 is normed for individuals aged 2.5 to 90+ years. In this assessment, the examiner says a word and asks the participant to point to the picture (out of a set of four) that shows the meaning of the word. The mean PPVT-4 SS for the general population is 100 (SD = 15). The lowest possible SS is 20.

#### Expressive Vocabulary Ability

Expressive vocabulary ability was measured by the Expressive Vocabulary Test-2 (EVT-2; Williams, 2007), which was co-normed with the PPVT-4. The EVT-2 is normed for individuals aged 2.5 to 90+ years. Most items require the participant to name the object or action depicted in the picture. For some items, the participant is shown a picture and asked to provide a synonym for a word produced by the examiner. The mean EVT-2 SS for the general population is 100 (SD = 15). For a child aged 48 months, the lowest possible SS is 25.

#### Intellectual Abilities

Intellectual abilities were measured by the Mullen Scales of Early Learning (MSEL; Mullen, 1995). The MSEL includes four scales: Visual Reception (measuring primarily nonverbal reasoning), Fine Motor (measuring primarily visuospatial construction), Receptive Language, and Expressive Language. Overall intellectual abilities were determined by the Early Learning Composite (ELC) which is based on performance on all four scales. The mean MSEL-ELC for the general population is 100 (SD = 15). The lowest possible MSEL-ELC is 49.

### Procedure

At age 48 months, all participants were tested at the Neurodevelopmental Sciences Laboratory at the University of Louisville as part of an ongoing longitudinal study of the early language and cognitive abilities of children with WS. Children completed the MSEL, PPVT-4, and EVT-2 following standardized procedures as part of a larger 2-day assessment. The first 48-month assessment was completed in May 2000 (when the first participant turned 48 months old) and the last in December 2018. Assessments completed beginning in April 2007 (when the first participant to turn 48 months after the PPVT-4 and EVT-2 were published had her assessment) included these two measures as well.

Parents completed the CDI as part of their child’s assessment at age 48 months; data from this administration were used to determine 48-month CDI-EV and 48-month CDI-SC. In addition, once children were enrolled in the study (typically by age 18 months) parents provided monthly updates on the CDI-W&G Early Communicative Gestures Checklist, the CDI-W&S Vocabulary Checklist, and the CDI-W&S Early Sentence Checklist either *via* phone interview or during visits to the laboratory. The second author conducted almost all of these monthly updates; of the updates she did not complete, most were administered by the first author. The mean number of monthly updates was 29.19 (SD = 4.08). The data from these monthly updates were used to determine the age of onset of declarative *show* and declarative *point* and CDI-EV at 24 months, at the production of first spontaneous referential word, and at the time of onset of each gesture.

### Data Analysis

Data were analyzed using IBM SPSS v. 25. The distributions of ages at onset of declarative *show* and declarative *point* and the distributions of CDI-EV at onset of *show* and *point* were non-normal. For this reason, nonparametric Wilcoxon related-samples signed rank tests were used to determine if there were significant differences in median age of onset of the two declarative gestures and to determine if there were significant differences in median CDI-EV at the time of onset of each of these gestures. Effect size was measured as *r* (0.1 = small effect, 0.3 = medium, 0.5 = large).

Pearson correlations were used to compute bivariate relations among the independent and dependent variables included in the regression analyses. For the correlational analyses, *α* was set at *p* = 0.01, two-tailed.

A series of multiple regression analyses was performed to predict vocabulary abilities (CDI-EV, PPVT-4 SS, and EVT-2 SS), grammatical abilities (CDI-SC), and overall intellectual ability (MSEL-ELC) at 48 months. For each analysis, the independent variables were CA at onset of declarative pointing gestures and CDI-EV at 24 months. Each independent variable was centered on the sample mean, which resulted in the constant being very close to the sample mean for the dependent variable, making the constant readily interpretable. The forced-entry method (“Enter” in SPSS) was used with both independent variables entered in a single block. The 95% confidence intervals are reported for all *B*-values, as are semi-partial correlations between the dependent variable and each of the two independent variables. Effect size was measured by Cohen’s *f*^2^ (0.02 = small effect, 0.15 = medium, 0.35 = large).

## Results

### Age at Onset of Declarative *Show* and *Point* and Relations to Expressive Vocabulary

Descriptive statistics for participants’ CA at onset of declarative *show* and *point* are presented in Table 1. All but one of the participants had begun to produce declarative *show* by age 60 months, and all but five participants (one of whom was the child who had not produced declarative *show* by 60 months) had begun to produce declarative *point* by age 60 months. For two of these five children, data collection continued beyond age 60 months, and the actual age of onset of declarative *point* was known. For the three remaining children (including the child who had not produced declarative *show* by 60 months), we coded age of onset of these declarative gestures as 61 months and their CDI-EV at gesture onset as their CDI-EV at the last time their mother completed the CDI-W&S prior to age 61 months. These decisions likely underestimated both their CA and their CDI-EV at declarative gesture onset but allowed these children, who were among the most delayed in the sample, to be included in the analyses.

**Table 1:** Descriptive statistics for chronological age at onset of declarative *show* and *point*.

Gesture	Chronological age in months at gesture onset				
	Mean	Median	SD	Interquartile range	Range
*Show*	23.23	20.86	9.10	17.58–25.07	13.21–61.00
*Point*	29.27	24.08	13.60	20.40–31.87	14.55–63.54

*N = 47*.

To determine if there were significant differences in median CA at onset of declarative *show* and *point*, a related-samples Wilcoxon signed rank test was performed. Results indicated that median CA at onset of *show* was significantly younger than median CA at onset of *point*, *z* = 5.02, *p* < 0.001, *r* = 0.51. Of the 47 children, 36 (76.6%) began to produce declarative *show* before beginning to produce declarative *point*, 7 (14.9%) began to produce both declarative gestures in the same month, and 4 (8.5%) began to produce declarative *point* prior to producing declarative *show*.

To determine the timing of onset of declarative gestures relative to onset of referential expressive vocabulary for children with WS, we compared CA at onset of each declarative gesture to CA at production of first referential word. The findings are presented in Table 2. As shown in Table 2, 72% of the children began to produce referential language prior to producing declarative *show* (median interval = 2.00 months, IQR: 0.00–5.65 months), and 89% began to produce referential language prior to producing declarative *point* (median interval = 7.10 months, IQR: 3.02–12.75 months).

**Table 2:** Number and percentage of children (*N* = 47) who evidenced various relations between age at the onset of declarative gestures and age at the onset of referential expressive language.

Declarative gesture	Relation between gesture and expressive language onsets					
	Gesture onset prior to referential expressive language onset		Gesture onset at same age as referential expressive language onset		Gesture onset after referential expressive language onset	
	*N*	%	*N*	%	*N*	%
*Show*	9	19.2	4	8.5	34	72.3
*Point*	2	4.2	3	6.4	42	89.4

Descriptive statistics for CDI-EV at onset of declarative *show* and *point* are presented in Table 3. To determine if there were significant differences in median CDI-EV at onset of the two declarative gestures, a Wilcoxon test was conducted. Results indicated that median CDI-EV at onset of declarative *show* was significantly smaller than median CDI-EV at onset of declarative *point*, *z* = 4.83, *p* < 0.001, *r* = 0.50.

**Table 3:** Descriptive statistics for CDI-EV at onset of declarative *show* and *point*.

Gesture	CDI-EV at declarative gesture onset^1^				
	Mean	Median	SD	Interquartile range	Range
*Show*	7.79	6.00	9.90	1.00–12.00	0–60
*Point*	36.62	14.00	52.73	7.00–41.00	0–246

*CDI-EV, MacArthur-Bates Communicative Development Inventory: Words and Sentences expressive vocabulary size*.

*N = 47*.

1 *If the child had no referential words in his/her expressive vocabulary at declarative gesture onset, then CDI-EV was considered to be 0*.

Children were not assessed for ASD as part of the present study. At the start of the study, none of the participants had a diagnosis of ASD. However, due to gradually emerging concerns regarding socio-communicative development, parents of several participants subsequently had their child formally assessed for ASD either by other researchers or by clinicians. By age 5 years, nine children (19.1%) had been diagnosed with ASD based on performance on the Autism Diagnostic Observation Schedule (ADOS; Lord et al., 1999) or the ADOS-2 (Lord et al., 2012) combined with the clinical judgment of a licensed psychologist who had extensive experience with both children with ASD and children with WS. Two children were diagnosed during the present study; the other seven were diagnosed after the present study ended. For the children diagnosed with ASD, CDI-EV at 48 months ranged from 4 to 395 words. All nine children produced referential words prior to age 48 months. Of the nine children with ASD, two produced declarative *show* and none produced declarative *point* prior to producing their first referential word. The six children in the full sample who had the longest intervals between production of first referential word and onset of declarative *point* (range: 23.88–39.94 months) all were diagnosed with ASD. However, the other three children with ASD evidenced a much smaller interval between production of first referential word and onset of declarative *point* (1.94–8.93 months). This interval was shorter than the median interval for the full sample (7.10 months) for two of the three children and well within the IQR for the third child. These three children also had considerably larger CDI-EVs at 48 months (166–395 words) than the other six children with ASD (4–34 words).

### Descriptive Statistics and Correlations Among Variables Included in the Regression Analyses

Descriptive statistics for the variables included in the regression analyses are presented in Table 4. Performance on each of these measures was characterized by considerable variability. For measures for which norms were available, performance clearly was below expectations for CA. Based on the CDI-EV norms for 24-month-olds, 37 of 47 participants (78.7%) scored below the 5th percentile (the lowest percentile included in the norms), 4 (8.5%) scored at the 5th percentile, 2 (4.3%) at the 10th percentile, 2 (4.3%) at the 15th percentile, and 2 (4.3%) at the 25th percentile. Mean PPVT-4 SS, EVT-2 SS, and MSEL-ELC were within two points of the means reported by Mervis and John (2010) for large groups of children with WS, suggesting that the present sample is likely representative of children with WS.

**Table 4:** Descriptive statistics for variables included in the regression analyses.

Variable	Mean	Median	SD	Interquartile range	Range
**Independent**					
CDI-EV at 24 months	43.21	22.00	41.89	4.00–41.00	0–176
CA (in months) at onset of declarative pointing	29.27	24.08	13.60	20.40–31.87	14.55–63.54
**Dependent**					
CDI-EV at 48 months	388.72	423.00	220.93	196.00–569.00	4–679
CDI-SC at 48 months	14.91	9.00	14.44	0–31.00	0–37^a^
PPVT-4 SS at 48 months	80.08	89.00	24.50	65.50–97.00	20^b^–113
EVT-2 SS at 48 months	79.62	83.00	23.32	71.50–97.00	25^c^–111
MSEL-ELC at 48 months	63.40	61.00	13.60	50.00–75.00	49^b^–96

*CDI-EV, MacArthur-Bates Communicative Development Inventory: Words and Sentences expressive vocabulary size; CA, chronological age; CDI-SC, MacArthur-Bates Communicative Development Inventory: Words and Sentences sentence complexity score; PPVT-4, Peabody Picture Vocabulary Test-4; EVT-2, Expressive Vocabulary Test-2; MSEL-ELC, Mullen Scales of Early Learning Early Learning Composite; SS, standard score*.

*N = 47 for all measures except for PPVT-4 SS and EVT-2 SS, for which *N* = 37*.

a *highest possible CDI-SC*;

b *lowest possible SS*;

c *lowest possible SS for 48-month-olds*.

Pearson correlations among the variables included in Table 4 are presented in Table 5. All correlations were significant. The strongest correlations (all *r*s > 0.90) were among the three measures of vocabulary at 48 months (CDI-EV at 48 months, PPVT-4 SS, and EVT-2 SS). Notably, the weakest correlation was between CDI-EV at 24 months and CA at onset of declarative *point*. CA at onset of declarative *point* was significantly more strongly correlated with CDI-EV at 48 months than with CDI-EV at 24 months (*z* = 3.73, *p* < 0.001).

**Table 5:** Bivariate correlations among the variables included in the regression analyses.

Measure	2	3	4	5	6	7
1. CDI-EV at 24 months	−0.46^*^	0.64^**^	0.64^**^	0.61^**^	0.65^**^	0.76^**^
2. CA at onset of declarative pointing		−0.79^**^	−0.60^**^	−0.84^**^	−0.81^**^	−0.59^**^
3. CDI-EV at 48 months			0.88^**^	0.93^**^	0.92^**^	0.82^**^
4. CDI-SC at 48 months				0.81^**^	0.77^**^	0.84^**^
5. PPVT-4 SS at 48 months					0.95^**^	0.78^**^
6. EVT-2 SS at 48 months						0.78^**^
7. MSEL-ELC at 48 months						

*CDI-EV, MacArthur-Bates Communicative Development Inventory: Words and Sentences expressive vocabulary size; CA, chronological age; CDI-SC, MacArthur-Bates Communicative Development Inventory: Words and Sentences sentence complexity score; PPVT-4, Peabody Picture Vocabulary Test-4; EVT-2, Expressive Vocabulary Test-2; MSEL-ELC, Mullen Scales of Early Learning Early Learning Composite; SS, standard score*.

* *p < 0.01*,

** *p < 0.001*.

### Multiple Regressions: Contributions of Early Lexical and Gestural Abilities to Language and Intellectual Abilities at 48 Months

To identify the contributions of CDI-EV at 24 months and CA at onset of declarative *point* to lexical, grammatical, and intellectual abilities at 48 months, a series of multiple regressions was performed. Dependent variables included language abilities as measured by the CDI-W&S and vocabulary and overall intellectual abilities as measured by standardized assessments.

#### Lexical and Grammatical Abilities as Measured by the MacArthur-Bates Communicative Development Inventory: Words and Sentences

To determine the contributions of individual differences in CDI-EV at 24 months and CA at onset of declarative *point* to later lexical (CDI-EV) and grammatical (CDI-SC) abilities, two multiple regressions were performed. All 47 children were included in the analyses.

The dependent variable for the first multiple regression was CDI-EV at 48 months. The model including CDI-EV at 24 months and CA at onset of declarative *point* accounted for a very large amount of variance in CDI-EV at 48 months, *R*^2^ = 0.72, adjusted *R*^2^ = 0.71, *F*(2,44) = 56.69, *p* < 0.001. As indicated in Table 6, both predictors accounted for a significant amount of variance. The effect size was medium to large for CDI-EV at 24 months and large for CA at onset of declarative *point*. After controlling for the effects of CA at onset of declarative *point*, a one-word increase in CDI-EV at 24 months resulted in an 1.83-word increase in CDI-EV at 48 months. After controlling for the effect of CDI-EV at 24 months, a 1-month increase in CA at onset of declarative *point* resulted in a 10.25-word decrease in CDI-EV at 48 months. CDI-EV at 24 months uniquely accounted for 9.6% of the variance in CDI-EV at 48 months, and CA at onset of declarative *point* uniquely accounted for 31.4% of the variance.

**Table 6:** Multiple regression analyses predicting language abilities as measured by the MacArthur-Bates Communicative Development Inventory: Words and Sentences.

Predictor	*B*	*t*	*p*	95% CI for *B*	Semi-partial *r*	Cohen’s ƒ^2^
**CDI-EV at 48 months**						
Constant	388.76	22.31		[353.64 to 423.87]		
CDI-EV at 24 months	1.83	3.88	<0.001	[0.88 to 2.79]	0.31	0.34
CA at onset of declarative pointing	−10.25	−7.04	<0.001	[−13.19 to −7.32]	−0.56	1.13
**CDI-SC at 48 months**						
Constant	14.91	10.07	<0.001	[11.93 to 17.90]		
CDI-EV at 24 months	0.16	3.96	<0.001	[0.08 to 0.24]	0.41	0.35
CA at onset of declarative pointing	−0.41	−3.32	0.002	[−0.66 to −0.16]	−0.34	0.25

*CA, chronological age in months; CDI-EV, MacArthur-Bates Communicative Development Inventory: Words and Sentences expressive vocabulary size; CDI-SC, MacArthur-Bates Communicative Development Inventory: Words and Sentences sentence complexity score*.

*N = 47*.

*Both independent variables were centered on the sample mean*.

The dependent variable in the second multiple regression analysis was CDI-SC at 48 months. The combination of CDI-EV at 24 months and CA at onset of declarative *point* accounted for a large amount of variance in CDI-SC, *R*^2^ *= 0.*52, adjusted *R*^2^ = 0.51, *F*(2,44) = 24.45, *p* < 0.001. As indicated in Table 6, both predictors accounted for a significant amount of the variance. The effect size was large for CDI-EV at 24 months and medium for CA at onset of declarative *point*. After controlling for CA at onset of declarative *point*, a one-word increase in CDI-EV at 24 months resulted in a 0.16-item increase in CDI-SC. After controlling for CDI-EV at 24 months, a 1-month increase in CA at onset of declarative *point* accounted for a 0.41-item decrease in CDI-SC at 48 months. CDI-EV at 24 months uniquely accounted for 16.8% of the variance in CDI-SC at 48 months, and CA at onset of declarative *point* uniquely accounted for 11.6% of the variance.

#### Lexical Abilities as Measured by Standardized Assessments

Two multiple regressions were performed to determine the contributions of individual differences in CDI-EV at 24 months and CA at onset of declarative *point* to later receptive and expressive vocabulary abilities as measured by standardized assessments. The 37 children in the sample who turned 48 months old after the PPVT-4 and EVT-2 were published were included in the analyses.

The dependent variable for the first multiple regression analysis was PPVT-4 SS at 48 months. One child was excluded from the analysis because his standardized residual was 3.52 SD below the sample mean. The model accounted for a very large amount of variance, *R*^2^ *= 0*.77, adjusted *R*^2^ = 0.75, *F*(2,33) = 54.10, *p* < 0.001. As indicated in Table 7, both predictors accounted for a significant amount of the variance, with a medium effect size for CDI-EV at 24 months and a large effect size for CA at onset of declarative *point*. After controlling for the effect of CA at onset of declarative *point*, a one-word increase in CDI-EV at 24 months resulted in a 0.14-point increase in PPVT-4 SS at 48 months. After controlling for CDI-EV at 24 months, a 1-month increase in CA at onset of declarative *point* resulted in a 1.34-point decrease in PPVT-4 SS at 48 months. CDI-EV at 24 months uniquely accounted for 5.6% of the variance in PPVT-4 SS and CA at onset of declarative *point* uniquely accounted for 39.7%.

**Table 7:** Multiple regression analyses predicting language abilities as measured by standardized single-word vocabulary assessments.

Predictor	*B*	*t*	*p*	95% CI for *B*	Semi-partial *r*	Cohen’s ƒ^2^
**PPVT-4 SS at 48 months**						
Constant	75.45	40.31	<0.001	[75.44 to 83.46]		
CDI-EV at 24 months	0.14	2.78	0.009	[0.04 to 0.24]	0.23	0.23
CA at onset of declarative pointing	−1.34	−7.44	<0.001	[−1.71 to −0.97]	−0.63	1.68
**EVT-2 SS at 48 months**						
Constant	77.40	39.10	<0.001	[73.38 to 81.42]		
CDI-EV at 24 months	0.18	3.59	0.001	[0.08 to 0.28]	0.31	0.34
CA at onset of declarative pointing	−1.22	−6.74	<0.001	[−1.58 to −0.85]	−0.58	1.34

*CA, chronological age in months; CDI-EV, MacArthur-Bates Communicative Development Inventory: Words and Sentences expressive vocabulary size; PPVT-4, Peabody Picture Vocabulary Test-4; EVT-2, Expressive Vocabulary Test-2; SS, standard score*.

*N = 36 for PPVT-4 SS, *N* = 37 for EVT-2 SS*.

*Both independent variables were centered on the sample mean*.

The second multiple regression analysis in this set considered EVT-2 SS at 48 months as the dependent variable. The model accounted for a very large amount of variance, *R*^2^ *= 0*.75, adjusted *R*^2^ = 0.74, *F*(2,34) = 51.50, *p* < 0.001. As indicated in Table 7, both predictors accounted for a significant amount of unique variance, with a moderate to large effect size for CDI-EV at 24 months and a large effect size for CA at onset of declarative *point*. After controlling for the effect of CA at onset of declarative *point*, a one-word increase in CDI-EV at 24 months resulted in a 0.18-point increase in EVT-2 SS at 48 months. After controlling for the effect of CDI-EV at 24 months, a 1-month increase in CA at onset of declarative *point* resulted in a 1.22-point decrease in EVT-2 SS. CDI-EV at 24 months uniquely accounted for 9.6% of the variance in EVT-2 SS and CA at onset of declarative *point* uniquely accounted for 33.6%.

#### Overall Intellectual Ability as Measured by the Mullen Scales of Early Learning-Early Learning Composite

A multiple regression analysis was performed to determine the contributions of individual differences in CDI-EV at 24 months and CA at onset of declarative *point* to later overall intellectual ability. For this analysis, MSEL-ELC at 48 months was the dependent variable. All 47 participants were included in the analysis. The model accounted for a large amount of variance, *R*^2^ = 0.64, adjusted *R*^2^ = 0.63, *F*(2,44) = 39.78, *p* < 0.001. As indicated in Table 8, both predictors accounted for a significant amount of unique variance in MSEL-ELC. The effect size was large for CDI-EV at 24 months and medium for CA at onset of declarative *point*. After controlling for the effects of CA at onset of declarative *point*, a one-word increase in CDI-EV at 24 months resulted in a 0.20-point increase in MSEL-ELC. After controlling for the effect of CDI-EV at 24 months, a 1-month increase in CA at onset of declarative *point* resulted in a 0.31-point decrease in MSEL-ELC. CDI-EV at 24 months uniquely accounted for 30.2% of the variance in MSEL-ELC at 48 months, and CA at onset of declarative *point* uniquely accounted for 7.3% of the variance^1^.

**Table 8:** Multiple regression analysis predicting overall intellectual abilities as measured by the MSEL-ELC.

Predictor	*B*	*t*	*p*	95% CI for *B*	Semi-partial *r*	Cohen’s ƒ^2^
Constant	63.41	52.38	<0.001	[60.97 to 65.84]		
CDI-EV at 24 months	0.20	6.08	<0.001	[0.13 to 0.27]	0.55	0.84
CA at onset of declarative pointing	−0.31	−3.02	0.004	[−0.51 to −0.10]	−0.27	0.21

*CA, chronological age; CDI-EV, MacArthur-Bates Communicative Development Inventory: Words and Sentences expressive vocabulary size; MSEL-ELC, Mullen Scales of Early Learning Early Learning Composite*.

*Both independent variables were centered on the sample mean*.

## Discussion

As expected based on the findings of Mervis and Bertrand (1997), most toddlers with WS began to produce referential language prior to beginning to produce declarative pointing or showing gestures. This sequential order is the opposite of that shown by most TD infants. Nevertheless, the age at which toddlers with WS began to produce declarative pointing gestures was a strong predictor of individual differences in a variety of vocabulary measures at 48 months, even after accounting for the contribution of 24-month expressive vocabulary. In fact, the declarative gesture measure accounted for considerably more unique variance in 48-month measures of vocabulary than did 24-month expressive vocabulary. This pattern – in contrast to the pattern for the relation between the onset of declarative gestures and the onset of referential expressive language – is consistent with prior findings for TD children. The declarative gesture measure also contributed significant unique variance beyond that contributed by 24-month expressive vocabulary to 48-month grammatical ability and overall intellectual ability, although for these measures, the declarative gesture measure contributed less unique variance than the vocabulary measure. In the remainder of the Discussion, we consider these findings in the context of prior research on both TD children and children with DD.

### Onset of Declarative *Point* and *Show* and Relations to Onset of Expressive Referential Language

The first aim of our study was to determine the temporal relation between the onset of declarative gestures and the onset of referential expressive language for toddlers with WS. We found that the onset of declarative gestures was considerably delayed. In contrast to the median age of onset of 8 months for declarative *show* and 10 months for declarative *point* for the TD infants in the norming sample for the CDI (Fenson et al., 1994), for toddlers with WS the median ages of onset, based on the same measure, were more than 12 months older (21 months for declarative *show* and 24 months for declarative *point*). These delays are considerably larger than the delay for onset of expressive referential language, which occurs after the onset of declarative *point* for TD infants (e.g., Carpenter et al., 1998), but a median of 7 months before the onset of declarative *point* for the toddlers with WS in the present study. The finding that the onset of referential expressive language preceded the onset of declarative *point* for 89% of the toddlers with WS in the present sample replicates Mervis and Bertrand’s (1997) finding that 90% of an independent and considerably smaller sample of children with WS began to produce referential language prior to comprehending or producing declarative pointing gestures. This pattern contrasts strongly with that both for TD infants and for toddlers with a variety of other DDs (see review in Mervis and Bertrand, 1997) but fits with Talbott et al.’s (in press) finding that in a sample of children with ASD, only 38% produced distal pointing gestures prior to the onset of expressive language. As discussed in the next section, this delay in the onset of referential gestures likely is an important contributor to the language delay associated with WS. This is because caregivers are significantly more likely to label an object if the child has just pointed to it referentially than if the child has vocalized or reached for the object (Masur, 1982; Wu and Gros-Louis, 2015; Dimitrova et al., 2016). This timing provides the child with the name for the object at a time when it is the focus of his/her attention.

None of the children in the present sample had been diagnosed with ASD prior to the start of the study. However, by age 5 years, 19% had received this diagnosis based on gold-standard assessment. Although the prevalence of ASD among children with WS has not been determined definitively, this percentage is consistent with the 20% rate reported in the only study of children with WS that included a clinical diagnosis component in addition to administration of the ADOS (Lincoln et al., 2007). Considerable overlap in socio-communication difficulties between children with ASD and children with WS has been found even for children with WS who likely do not have ASD (Klein-Tasman et al., 2007, 2018). Difficulty with comprehension and production of communicative gestures is one such area (Laing et al., 2002; John and Mervis, 2010; Klein-Tasman et al., 2018).

### Contributions of Early Gestural and Lexical Abilities to Later Language and Intellectual Abilities

Our second aim was to determine the contributions of age of onset of declarative *point* and expressive vocabulary size at 24 months to the child’s lexical, grammatical, and overall intellectual abilities at 48 months. Both independent variables contributed significant amounts of unique variance to individual differences in each of the five outcome measures. Below we consider first the findings for the lexical outcome measures and then the findings for the grammatical and overall intellectual ability outcome measures.

#### Lexical Outcome Measures

For the three lexical outcome measures, individual differences in performance on the two independent variables accounted for a very large amount of variance, with adjusted *R*^2^ values ranging from 0.71 to 0.75. Strikingly, for each of these outcome measures, age of onset of declarative *point* accounted for considerably more unique variance than did 24-month expressive vocabulary size: more than three times for the two measures of expressive vocabulary (CDI-EV and EVT-2 SS) and more than seven times for the measure of receptive vocabulary (PPVT-4 SS).

This pattern of stronger contribution of early declarative gesture ability than early lexical ability to individual differences in later lexical ability is consistent with Rowe and Goldin-Meadow’s (2009) findings for TD children. In that study, the independent variables were the number of different gesture meanings (most of which were declarative; see Sauer et al., 2010) and the number of different word types as measured during a play session with a parent at age 18 months. Together, the independent variables accounted for 30.9% of the variance in the outcome measure, PPVT-III SS at 42 months, with each independent variable accounting for a significant amount of unique variance. Amount of unique variance accounted for by each independent variable was not reported, but *β* was at least nominally larger for the gesture measure than for the expressive vocabulary measure. In an earlier paper reporting on the same sample but with the independent variables measured at 14 months rather than 18 months, only the gesture measure accounted for a significant amount of unique variance (Rowe et al., 2008) in PPVT-III SS at 42 months. In the third study, Sauer et al. (2010) considered the contributions of the same independent variables measured at 18 months to individual differences in PPVT-III SS at 30 months for a sample of children with PL. Together, the independent variables accounted for 52.8% of the variance in PPVT-III SS, with only the gesture variable contributing a significant amount of unique variance. Both Rowe et al. (2008) and Sauer et al. (2010) noted that the lack of contribution of the earlier lexical measure to later PPVT-III SS in their studies likely was due to there being very little variation among children in expressive vocabulary size at the age at which it was measured.

As the present findings and those of Rowe and Goldin-Meadow (2009) indicate, when early lexical ability is measured at an age at which considerable individual differences are found, early lexical ability does make a significant contribution to individual differences in later lexical ability. Nevertheless, the contribution of early declarative gesture ability appears to be stronger. Why might declarative gestural ability at a younger age contribute more unique variance than early lexical ability to later lexical ability, even when there is considerable variability in the measure of early lexical ability?

One possibility is that the child’s use of declarative gestures leads to changes in the language of the child’s interactive partners. For example, adults might be more likely to label an object if the child had just pointed to it. This, in turn, would provide the child with a label for the object when it was the focus of his or her attention, increasing the likelihood that the child would benefit from the adult’s input and learn to comprehend the object’s name. Findings consistent with this possibility include that mothers of TD children were more likely to label the referent of their child’s focus of attention if the child had pointed to the object than if the child had reached for it (Masur, 1982) or had vocalized while looking at the object with or without gaze alternation to the adult (Wu and Gros-Louis, 2015) and that mothers of TD children, children with ASD, and children with DS responded verbally to more than 90% of their children’s declarative gestures, with about three-quarters of these responses including the object’s name (Dimitrova et al., 2016). Furthermore, both TD children (Goldin-Meadow et al., 2007; Dimitrova et al., 2016) and children with ASD or DS (Dimitrova et al., 2016) were significantly more likely to later produce the name of an object whose name was not originally in their lexicon if the mother had labeled the object in response to the child’s pointing gesture than if the child had pointed to the object but the mother had not responded by labeling it.

For children with DD, a second important consideration is that speech/language therapists and other developmental therapists often consider the onset of declarative gesture production as the primary indicator that the child would benefit from language therapy; it is at this point that many therapists begin to focus on vocabulary acquisition rather than on prelinguistic skills (Mervis and Becerra, 2007; Mervis and Velleman, 2011). Once therapists begin to focus on vocabulary development, parents also may change their input to put more emphasis on the provision of object labels contingent on the child’s focus of attention, whether the child has indicated that focus by producing a declarative gesture or by looking at an object or manipulating the object without gesturing toward it.

Given the contribution of early declarative gesture production to later vocabulary acquisition, early intervention designed to facilitate the onset of spontaneous use of declarative gestures in toddlers with WS and to increase the frequency of these gestures by toddlers with WS who rarely produce these gestures is likely to be beneficial. This intervention should begin before the child starts to produce spontaneous expressive language and can be concurrent with other interventions designed to facilitate vocabulary acquisition. Given prior meta-analytic findings for both TD children and children with ASD that declarative gesture frequency but not imperative gesture frequency is related to later vocabulary development, the focus should not be on facilitating gesture production in general but rather on facilitating truly declarative gestures (those that reflect interest in the object and eagerness to share attention regarding it). Harbison et al. (2017) provide a brief review of strategies that have led to successful acquisition and generalization of declarative gestures by children with ASD; many of these are likely to be helpful for children with WS as well.

#### Grammatical and Overall Intellectual Ability Outcomes

Individual differences in age of onset of declarative point and 24-month CDI-EV each contributed significant unique variance to both grammatical complexity and overall intellectual ability at 48 months, accounting for a large amount of variance in the dependent variables (adjusted *R*^2^ = 0.51 for CDI-SC and 0.63 for MSEL-ELC). However, the relative importance of the two independent variables was different from that found for the lexical outcome measures. For CDI-SC, individual differences on the two independent variables contributed approximately equal amounts of unique variance, and for MSEL-ELC, 24-month CDI-EV contributed four times as much unique variance as age at onset of declarative *point*.

The finding that age at onset of declarative *point* contributed considerably less unique variance to 48-month grammatical complexity than to lexical ability at the same age fits with Rowe and Goldin-Meadow’s (2009) finding that the 18-month gesture measure (number of different meanings expressed) that predicted 42-month spoken vocabulary for TD children did not predict 42-month grammatical complexity. Instead, a different 18-month gesture measure did: gesture + speech combinations in which the gesture indicated one idea and the word indicated a different idea. The age of onset of these types of gesture-speech combinations for TD toddlers had previously been shown to predate and correlate strongly with the age at onset of two-word combinations (Iverson and Goldin-Meadow, 2005).

We are not aware of previous studies that have considered the relation between very early lexical or gestural abilities and later intellectual abilities. It is possible that as children get older, language ability plays an increasing role not only in verbal but also in non-verbal intellectual ability; although by definition, non-verbal tasks can be solved without language, their solution often is facilitated by verbal mediation. It is also possible that early gestural ability is an important predictor of later intellectual ability, but a different measure of early gestural ability is needed rather than the simple measure we used.

### Limitations

In this study, our independent variables were both based on parental report rather than on direct observation during either a parent-child play session or a more structured activity such as the CSBS. The use of parental report allowed us to collect monthly data for a relatively large sample of children with a rare genetic disorder; given the prevalence of WS, this would not have been feasible if monthly laboratory visits or monthly trips by researchers to the children’s homes had been required. Considerable care was taken during the monthly data collection to make sure that mothers understood what was meant by the CDI declarative gesture questions and what constituted spontaneous use either of a single word (CDI-EV) or a particular grammatical construction (CDI-SC). Children were tested in the lab at age 48 months, with the majority of families traveling from a considerable distance to participate. Our finding that the correlations between 48-month expressive vocabulary ability as measured by parent report (CDI-EV) and vocabulary ability as measured by standardized assessment (PPVT-4 SS, EVT-2 SS) were extremely high (*r* = 0.92–0.93) and as high as the correlation between SSs on the two standardized vocabulary assessments provides support for the validity of our approach.

Our measure of declarative gesture ability – age at onset of declarative *point* – does not provide an indication of how often during a play session the child produced this type of gesture at a given age or the number of different meanings (types of objects referred to) that the child expressed with this type of gesture during the play session, which are the types of measure most commonly used in prior studies of TD toddlers or children with other DD. In this way, our findings are not directly comparable to those of prior studies. At the same time, our measure does provide a good indication of how long the child had been producing declarative gestures at age 48 months (or, for children who were not yet producing these gestures at that age, how far they were temporally from being able to produce them), which we did find was an important independent predictor of later language abilities. Because we were not able to measure gesture + speech combinations, we were not able to determine if age at onset or number of gesture-speech combinations in which the word conveyed a different idea from the gesture measured at a specific age was an independent predictor of later grammatical ability for children with WS, as it has been shown to be for TD children. This is an important question for future research.

## Conclusion

Most toddlers with WS begin to produce referential expressive language several months before they begin to produce declarative communicative gestures. This is the opposite order of onset from that shown by most TD children and children with a variety of other DDs, suggesting a divergence in the path to early language development for children with WS. This divergence does not indicate that the establishment of triadic joint attention is not important for children with WS to begin to produce referential language but rather that other means for establishing this attention are being used. In particular, it suggests that prior to the onset of referential language, the onus to establish triadic joint attention is predominantly on the communicative partners of children with WS, rather than this responsibility being more evenly shared between the child and communicative partner. In keeping with this argument, the age at which a child with WS began to produce declarative pointing gestures – a measure of how long the child has been able to produce this type of gesture and thus to take some responsibility for initiating triadic joint attention to referents of immediate interest to the child – is a stronger predictor of later lexical abilities than is early expressive vocabulary. Thus, similarly to what has been found for TD children and children with other DDs, production of declarative gestures referencing objects of particular interest likely leads to specific changes in the language input communicative partners provide to children with WS that facilitate their language development. In this way, despite the earlier divergence in their path to language development from the typical one, the path for children with WS re-converges with that for TD children and children with other DDs.

## Data Availability Statement

The datasets generated for this study are available on request to the corresponding authors.

## Ethics Statement

The studies involving human participants were reviewed and approved by the Institutional Review Board for Social and Behavioral Sciences at the University of Louisville. Written informed consent to participate in this study was provided by the participants’ parent or legal guardian.

## Author Contributions

CM and AB conceived and designed the research. CM collected almost all of the parental report data and AB collected most of the remaining parental report data. AB drafted the manuscript and CM and AB revised the manuscript and approved the final version.

### Conflict of Interest

The authors declare that the research was conducted in the absence of any commercial or financial relationships that could be construed as a potential conflict of interest.

## Footnotes

^1^The regression analyses also were conducted with only the children who were never diagnosed with ASD (*N* = 38). The basic findings remained the same: Both age at onset of declarative pointing and CDI-EV at 24 months made significant unique contributions to the variance in each of the five dependent variables.
